# Mindful diagnostics in culture-negative endocarditis: early noninvasive diagnosis of *Tropheryma whipplei* by plasma microbial cell-free DNA

**DOI:** 10.1017/ash.2026.10416

**Published:** 2026-06-16

**Authors:** Ashita Jain, Sarwat Khalil, Muhammad R. Sohail, Laura C. Lelenwa, Enrique Garcia-Sayan, Kenneth K. Liao, Ahmed Hamdi, Todd Lasco, Mayar Al Mohajer

**Affiliations:** 1 Baylor College of Medicine, Houston, TX, USA; 2 Nuffield Department of Primary Care, University of Oxford, Oxford, UK

## Abstract

*Tropheryma whipplei* was identified by plasma microbial cell-free DNA sequencing within 4 days in severe culture-negative endocarditis after antibiotics had already been started. The result supported earlier diagnostic clarification and antimicrobial de-escalation before valve pathology was available, illustrating a practical diagnostic-stewardship role for noninvasive sequencing in high-acuity endocarditis.

## Introduction

Culture-negative endocarditis requires urgent treatment despite incomplete microbiologic data. *Tropheryma whipplei* is an increasingly recognized cause, often presenting as isolated cardiac disease rather than classic gastrointestinal Whipple disease. Published series emphasize middle-aged men, aortic-valve involvement, and heart failure, and molecular studies suggest that *T. whipplei* is an important contributor to culture-negative endocarditis cohorts.^1–4^ Presentation may be dominated by cardiac manifestations, lowering clinical suspicion. Yet routine blood cultures are usually negative and blood polymerase chain reaction has limited sensitivity, so confirmation often depends on valve tissue obtained late in the clinical course.^2,3^ This delay can prolong exposure to broad-spectrum therapy and defer organism-directed management. In patients who receive empiric antibiotics before diagnostic sampling is complete, noninvasive molecular assays may offer earlier organism-directed guidance.^5^

## Clinical case

A 56-year-old man with heart failure presented with progressive lower-extremity edema, worsening fatigue, and 3 to 6 months of orthopnea. He was admitted to the medical intensive care unit with acute decompensation manifested by transaminitis, coagulopathy, elevated lactate, leukocytosis, thrombocytopenia, pleural effusions, acute kidney injury, and ascites. His history included hypertension, hyperlipidemia, and osteoarthritis. He denied injection drug use but reported remote tobacco use and occupational exposure to animals and animal feces.

**Figure 1.**
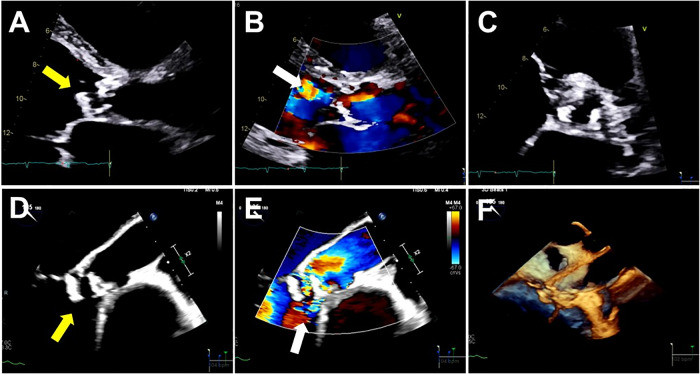
Transthoracic and transesophageal echocardiography of aortic-valve infective endocarditis. (A) Transthoracic parasternal long-axis view showing a large mobile aortic-valve vegetation (yellow arrow). (B) Color Doppler image showing severe eccentric aortic regurgitation (white arrow). (C) Additional transthoracic view demonstrating bicuspid aortic-valve morphology. (D) Transesophageal view showing a flail aortic leaflet with attached vegetation (yellow arrow). (E) Color Doppler transesophageal view confirming severe eccentric aortic regurgitation (white arrow). (F) Three-dimensional transesophageal echocardiography showing the abnormal bicuspid aortic valve.

Point-of-care ultrasonography showed an echogenic aortic-valve lesion. Transthoracic and transesophageal echocardiography demonstrated a dilated left ventricle with mildly reduced ejection fraction, bicuspid aortic-valve morphology, a large mobile vegetation, severe eccentric aortic regurgitation, and a flail leaflet (Figure 1).

Because infective endocarditis was strongly suspected, empiric ceftriaxone, meropenem, and vancomycin were started. Blood cultures were obtained only after the first ceftriaxone dose, potentially lowering culture yield. Plasma microbial cell-free DNA sequencing (Karius assay; Karius, Redwood City, CA) was collected on hospital day 2. Serologic and microbiologic evaluation for Brucella, Coxiella, Bartonella, and Lyme or rickettsial infection was unrevealing. Four days later, the assay detected a high burden of *T. whipplei*.

**Figure 2.**
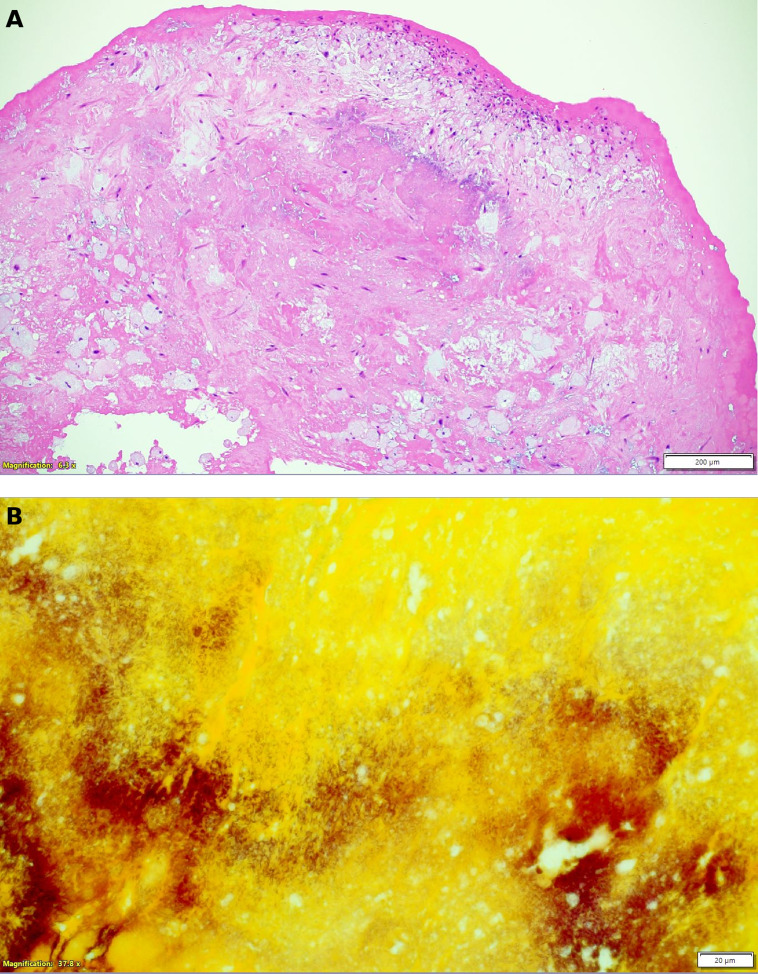
Surgical pathology of the excised valvular tissue. (A) Hematoxylin and eosin stain showing destruction of valvular tissue by vegetation, mixed inflammation, neovascularization, organizing fibrin thrombi, and calcification. (B) Gram stain at 60x magnification showing numerous gram-positive bacilli within the vegetation.

Therapy was narrowed to ceftriaxone after that result. Ten days after echocardiographic diagnosis, the patient underwent surgical aortic-valve replacement with a bovine pericardial valve and debridement and repair of infected tissue involving the aorto-mitral curtain. Surgical pathology showed destructive vegetation with mixed inflammation, neovascularization, organizing fibrin thrombi, and calcification; Gram stain demonstrated numerous gram-positive bacilli within the vegetation (Figure 2). Grocott-Gomori methenamine silver, Kinyoun acid-fast bacilli, and Fite stains were negative. He completed 4 weeks of ceftriaxone after surgery and then began a planned 18-month course of trimethoprim-sulfamethoxazole to reduce relapse risk. He was discharged home in stable condition nearly 3 weeks after admission and remained adherent to therapy at follow-up.

## A diagnostic stewardship framework for culture-negative endocarditis

This case suggests 3 stewardship questions when considering advanced diagnostics in culture-negative endocarditis. First, is the organism plausible in the clinical syndrome? *T. whipplei* deserves consideration in middle-aged men with culture-negative aortic-valve endocarditis, heart failure, or otherwise unexplained destructive valvular disease, even without diarrhea or other classic extracardiac features.^1–3^ The bicuspid aortic valve in this patient may also have increased susceptibility to endocarditis.

Second, will the result change management before invasive confirmation is available? Here, the patient was critically ill, and the blood cultures were likely compromised by antibiotic exposure. The plasma sequencing result supported the earlier discontinuation of meropenem and vancomycin and enabled narrower therapy before operative pathology was available. The assay was especially useful because invasive tissue confirmation was not immediately available. That earlier narrowing was clinically relevant because he presented with severe valvular dysfunction, heart failure, acute kidney injury, and multiorgan derangements.

Third, how should the test complement rather than replace standard evaluation? Plasma microbial cell-free DNA did not replace echocardiography, surgery, histopathology, or clinicopathologic correlation; instead, it provided microbiologic clarity when conventional blood culture was least informative.^5^ Published experience with plasma microbial cell-free DNA sequencing in *T. whipplei* endocarditis remains limited. In a focused review of the English-language literature, we identified 3 earlier reports, including 1 conference abstract and 2 full case reports.^6–8^ Across those reports and the present case, patients were men older than 50 years, aortic or mitral valve involvement predominated, and plasma sequencing established or strongly supported the diagnosis before or without valve-tissue confirmation. Our case presents with fulminant valvular dysfunction, prior antibiotic exposure, and subsequent pathology demonstrating abundant gram-positive bacilli.

## Conclusion

For ASHE readers, the central lesson is not that plasma microbial cell-free DNA should replace foundational endocarditis diagnostics. Rather, in carefully selected patients with suspected culture-negative endocarditis, especially after antibiotic exposure or when invasive sampling will be delayed, it can support earlier organism-directed therapy and reduce unnecessary broad-spectrum antimicrobial exposure while definitive tissue-based studies are pending.

## Data Availability

Deidentified clinical details supporting this case report are not publicly available because of patient privacy considerations.

## Artificial intelligence assistance

Artificial intelligence tools were used solely for minor editorial assistance related to grammar and language clarity. All content, analysis, and conclusions were developed by the authors.
